# 2024 ACVIM Forum Research Report Program

**DOI:** 10.1111/jvim.17177

**Published:** 2024-09-23

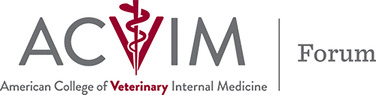

The American College of Veterinary Internal Medicine (ACVIM) Forum and the Journal of Veterinary Internal Medicine (JVIM) are not responsible for the content or dosage recommendations in the abstracts. The abstracts are not peer reviewed before publication. The opinions expressed in the abstracts are those of the author(s) and may not represent the views or position of the ACVIM. The authors are solely responsible for the content of the abstracts.

**2024 ACVIM Forum**

**June 5–October 31, 2024**

**Research Report Program**

**Index of Abstracts**

**Table:** 

**CARDIOLOGY**	
**Presenting Author**	**Abstract Title**
Brian Scansen	Assessment of Gastrointestinal Health in Dogs with Right‐Sided Heart Disease
Lance Visser	Comparing Echocardiographic Estimates of Stroke Volume in Healthy Dogs
Ashley Saunders	Comparison of Serologic Tests for Detecting *Trypanosoma cruzi* infection in Dogs
Joanna Kaplan	Establishing a Robust Genetic Sequencing and Gene Expression Data Library in Cardiovascularly Healthy Cats
**EQUINE**	
**Presenting Author**	**Abstract Title**
Gilliam Perkins	Antibody Testing to Detect Viral Exposure in Contact Horses During an Equine Herpes Myeloencephalopathy Outbreak
Breanna Sheahan	Comparison of Duodenal Biopsies and Organoids from Healthy Horses with and Without Insulin Resistance
Camilla Quattrini	Distribution of Alprazolam into the Milk of Lactating Mares and Subsequent Absorption by Nursing Foals
Elizabeth Williams Louie	Heart Rate Variability Associated with Endotoxin Administration in 13 Adult Horses
Laurent Couetil	Omega‐3 Polyunsaturated Fatty Acids Supplementation Modulates Airway Response to Dust Exposure in Thoroughbred Racehorses
**NEUROLOGY**	
**Presenting Author**	**Abstract Title**
Aude Castel	Alterations in Sensory and Motor Nerve Conduction Associated with Age and Osteoarthritis in Cats
Filippo Adamo	Biomechanical Study to Assess Stabilization of Adamo Spinal Disc™ in Cervical Disc Arthroplasty Using Tetranite®
Richard Shinn	Comparison of Stereotactic Brain Biopsy Techniques: Neuronavigation, 3D‐Printed Guides, and Neuronavigation with 3D‐Printed Guides
Fiona Inglis	Disposition of a Single Oral Dose of the Novel Antiseizure Drug Perampanel in Cats
Richard Shinn	Teleneurology in Veterinary Medicine for Neurolocalization
**NUTRITION**	
**Presenting Author**	**Abstract Title**
Robert Backus	Correlation Between Taurine Concentrations in Skeletal and Cardiac Muscle in Cadavers of Large Dogs
Sally Perea	Dietary Response of Dogs with Protein Losing Enteropathy: A Prospective, Randomized, Blinded Multicenter Clinical Study
**SMALL ANIMAL INTERNAL MEDICINE**	
**Presenting Author**	**Abstract Title**
Stacie Summers	Biological Variation of Serum Phosphorus and Fibroblast Growth Factor‐23 in Cats with Chronic Kidney Disease
Cooper Brookshire	Carbapenem‐Resistant Infections in Canine Patients: A Case Series
Danielle Rondeau	Comparison of Next‐Generation DNA Sequencing and Bacterial Culture in Bile from Dogs and Cats
Hilla Chen	Effects of Paricalcitol on Renal Secondary Hyperparathyroidism and Proteinuria in Dogs with CKD
Tarini Ullal	Evaluation of IL‐2 Suppression and Cyclosporine Concentrations in Dogs with Immune‐mediated Chronic Hepatitis
Tarini Ullal	Evaluation of Rhodanine‐stained Liver Cytology to Diagnose and Monitor Copper‐associated Chronic Hepatitis in Dogs
Chen Gilor	Exenatide Extended‐Release for Maintenance of Diabetic Remission in Cats
Shauna Blois	Immunophenotype and Interleukin‐17 Expression in Dogs with Immune Mediated Hematologic Disease
Giacomo Rossi	Neoplastic Transformation of the Colon Mucosa Correlates with an Altered Expression of RAAS in Dogs
Terza Brostoff	Oral Remdesivir and GS‐441524 Show Similar Efficacy in Treating Noneffusive Feline Infectious Peritonitis
Sara Jablonski	Prednisolone Pharmacokinetics in Dogs with Protein‐losing Enteropathy
JoAnn Morrison	Retrospective Investigation into Genetic Mutations Associated with Vaccine Adverse Events
Jane Sykes	Signalment Risk Factors for Coccidioidomycosis Test Positivity in 887 764 Dogs, 2012‐2023
Jennifer Granick	Survey of Antimicrobial Stewardship and Infection Prevention and Control Efforts Among US/Caribbean Veterinary Schools
Albert Jergens	Ultrastructural Changes in Colonic Mucosa of Dogs with Chronic Inflammatory Enteropathy Treated with Synbiotics
Jodi Westropp	Urinary Cystine/creatinine Concentrations in Dogs with Suspected Androgen‐Dependent Cystine Urolithiasis Before and After Castration

## CARDIOLOGY

## Assessment of Gastrointestinal Health in Dogs with Right‐Sided Heart Disease

### **Brian A. Scansen** ^1^; Sara Jablonski^2^; Sarah Shropshire^3^

#### ^1^Colorado State University, Fort Collins, CO, USA; ^2^Small Animal Clinical Sciences, Michigan State University, East Lansing, MI, USA; ^3^Clinical Sciences, Colorado State University, Fort Collins, CO, USA

**BACKGROUND:** Right‐sided heart disease (RHD) results in systemic venous hypertension. The clinical impact of elevated venous pressures on gastrointestinal health is unknown.

**HYPOTHESIS:** Measures of gastrointestinal dysfunction increase with increasing severity of RHD.

**ANIMALS:** Eighteen dogs with RHD: compensated RHD without hepatic venous dilation (Group 1), compensated RHD with hepatic venous dilation (Group 2), and decompensated RHD with ascites (Group 3).

**METHODS:** Prospective clinical trial. All dogs had a comprehensive echocardiogram performed, including evaluation of hepatic/caval vein distension and collapsibility. Beyond conventional hematologic, fecal, and urine testing, thromboelastography, N‐terminal pro‐B‐type natriuretic peptide (NT‐proBNP), C‐reactive protein (CRP), cobalamin, folate, methylmalonic acid (MMA), and serum intestinal fatty acid‐binding protein (I‐FABP) were measured. Differences between groups were compared by one‐way analysis of variance.

**RESULTS:** There were no differences between groups for cobalamin or folate. Three of 6 dogs in both Groups 1 and 2 showed hypercoagulability while 5 of 6 dogs in Group 3 showed hypocoagulability. Two Group 3 dogs had elevated CRP; CRP was normal in all other dogs. Both MMA (*P* =0.04) and I‐FABP (*P* =0.03) differed between groups, with values increasing from Group 1 to 3. NT‐proBNP progressively increased between groups (*P* <0.001) with mean (range) of 1168 pmol/L (493‐3016), 2918 (1125‐8117), and 5864 (3492‐10 000) respectively.

**CONCLUSIONS:** Gastrointestinal health is altered in RHD, with greater dysfunction present in decompensated heart failure. These findings may impact nutritional management of RHD. The impact of gastrointestinal health on absorption of nutrients or medications in dogs with RHD deserves further study.

## Comparing Echocardiographic Estimates of Stroke Volume in Healthy Dogs

### **Lance Visser**; Kate Davis; June Boon; Evan Ross; Jacqueline Sankisov; Abigail Laws

#### Colorado State University, Fort Collins, CO, USA

**BACKGROUND:** Differences in echocardiographic estimates of stroke volume (SV) calculated from different anatomic sites should theoretically be zero in a healthy dog but might vary due to technical issues, physiologic variability, or measurement error.

**HYPOTHESIS/OBJECTIVES:** We sought to compare SV at different anatomic sites that are used to calculate clinically important indices including shunt volume (ShuntVol) or regurgitant volume (RegVol) in a large sample of healthy dogs.

**ANIMALS:** Ninety healthy adult dogs.

**METHODS:** Prospective study. All dogs underwent an echocardiogram. Stroke volume was derived from the product of stroke distance and cross‐sectional area at the pulmonary valve (SVPV), aortic valve (SVAV), and mitral valve (SVMV) levels. Stroke volume was also derived from the difference of left ventricular end‐diastolic volume and end‐systolic volume using Simpson's method from a right parasternal long‐axis (SVLV_RPLx) and an apical 4‐chamber (SVLV_Ap4Ch) view. Bland–Altman plots and 95% reference intervals (Clinical Laboratory Standards Institute methodology) were generated.

**RESULTS:** Mean difference (95% limits of agreement) for ShuntVol (SVPV‐SVAV), RegVolLV_RPLx (SVLV_RPLx‐SVAV), RegVolLV_Ap4Ch (SVLV_Ap4Ch‐SVAV), and RegVolMV (SVMV‐SVAV) were −0.14 (−0.72, 0.44), −0.05 (−0.59, 0.48), −0.16 (−0.71, 0.39), and 0.12 (−0.76, 1.00) mL/kg, respectively, with all but RegVolLV_RPLx showing significant (*P* < .01) fixed bias. Reference intervals for ShuntVol, RegVolLV_RPLx, RegVolLV_Ap4Ch, and RegVolMV were −0.85‐0.64, −0.65‐0.58, −0.77‐0.52, and −0.91‐1.06 mL/kg, respectively.

**CONCLUSIONS AND CLINICAL IMPORTANCE:** Echocardiographic estimates of SV are not interchangeable and exhibit relatively wide limits of agreement. Reference intervals provide a frame of reference when quantitating lesion severity for dogs with a congenital shunt (ShuntVol) and mitral regurgitation (RegVol).

## Comparison of Serologic Tests for Detecting *Trypanosoma cruzi* Infection in Dogs

### **Ashley Saunders** ^1^; Sukjung Lim^1^; Sonya Gordon^1^; Victoria Miner^2^; Marty Henderson^3^; Kendra Zelachowski^1^; Samantha Eisner^1^; Sarah Hamer^1^; Brooke White^4^; Caleb Hawkins^4^; Rick Tarleton^4^

#### ^1^Texas A&M University, College Station, TX, USA; ^2^Boerne Veterinary Clinic, Boerne, TX, USA; ^3^SonoVet, New Braunfels, TX, USA; ^4^University of Georgia, Athens, GA, USA

**BACKGROUND:** Two positive serologic tests are recommended to confirm *T. cruzi* infection in humans. Serologic diagnosis remains challenging in dogs.

**OBJECTIVE:** To evaluate five serologic test results and blood PCR for *T. cruzi*.

**ANIMALS:** Eighteen client‐owned dogs.

**METHODS:** Observational, exploratory study evaluating two tests performed at reference/commercial laboratories including indirect fluorescent antibody (IFA) at Texas A&M Veterinary Medical Diagnostic Laboratory (TVMDL, College Station, TX) and ELISA at VRL Laboratories (San Antonio, TX), two point‐of‐care lateral flow immunochromatographic tests (Chagas STAT‐PAK validated for humans, Chembio Diagnostics; Vida Chagas for dogs, Vida Pharmacal), a multiplex microsphere immunoassay (Luminex), and PCR.

**RESULTS:** Dogs were tested based on clinical index of suspicion (3) or were asymptomatic living in high‐risk environments (15). Test results were positive for IFA in 83% (15/18), ELISA in 72% (13/18), STAT‐PAK in 39% (7/18), Vida in 45% (5/11), Luminex in 22% (4/18), and PCR in 17% (3/18). Luminex results provide a comprehensive assessment of responses to ~20 selected recombinant proteins identified as major targets of antibody responses in multiple species compared to a control, and positive results correlated with clinical disease. Additionally, the four Luminex positive dogs were the four with IFA titer >1280 and ELISA >3.0. Low positive IFA, ELISA, and STAT‐PAK results were associated with low reactivity to *T. cruzi* antigens that remained below the Luminex control.

**CONCLUSIONS AND CLINICAL IMPORTANCE:** Confirmation of single test results is recommended for low positive results and in asymptomatic dogs. Luminex results may help discriminate active *T. cruzi* infections in dogs.

## Establishing a Robust Genetic Sequencing and Gene Expression Data Library in Cardiovascularly Healthy Cats

### **Joanna L. Kaplan** ^1^; Victor Rivas^2^, MS, PHD Candidate; Jalena Wouters^3^; Joshua Stern^4^, DVM, PhD, DACVIM (Cardiology)

#### ^1^University of California‐Davis, Davis, CA, USA; ^2^Department of Clinical Sciences, North Carolina State University, Raleigh, NC, USA; ^3^Student Researcher, Department of Medicine and Epidemiology, University of California‐Davis, Davis, CA, USA; ^4^Associate Dean for Research and Graduate Studies, Department of Clinical Sciences, North Carolina State University, Raleigh, NC, USA

**BACKGROUND:** Cardiovascular disease is a leading cause of increased morbidity and mortality in cats, with hypertrophic cardiomyopathy (HCM) significantly overrepresented. While HCM is hereditary, the genetic etiology of disease remains poorly understood in the cat. Establishing a cohort of well‐phenotyped and ‐genotyped control cats is essential to fuel future genetic/pharmacogenetic discoveries.

**OBJECTIVES:** Develop a robust genetic sequencing and gene expression library from cardiovascularly healthy cats using whole genome sequencing (WGS) and RNA sequencing (RNA‐Seq).

**ANIMALS:** Fifty‐four apparently healthy client‐owned cats (≥10 years of age) and 14 purpose‐bred or client‐owned cats euthanized for non‐cardiac related causes.

**METHODS:** Blood samples from prospectively enrolled cats (Cohort1) were used to isolate DNA and subsequently submitted for paired‐end WGS at ~30X coverage. Standard variant calling pipelines were employed for variant calling across sequenced cats. Immediately flash‐frozen left ventricular (LV), interventricular septum (IVS), and left atrium (LA) tissue samples (Cohort2) were collected and submitted for stranded mature RNA‐Seq at 50 million reads/sample. All geriatric cats in this study were deemed true cardiovascularly healthy representatives of their species on clinicopathology, biochemistry, and echocardiography.

**RESULTS:** Of 54 cats screened in Cohort1, 19 cats were successfully enrolled. In Cohort2, flash‐frozen LV, IVS, and LA tissue from 11, 14, and 12 cats, respectively, underwent RNA‐Seq. Gene variants and expression profiling were catalogued for both meticulously selected cohorts.

**CONCLUSIONS:** Libraries of transcriptomic and WGS data in cardiovascularly normal cats were generated to serve as an open‐access resource in future investigations of feline cardiovascular medicine.

## EQUINE

## Antibody Testing to Detect Viral Exposure in Contact Horses During an Equine Herpes Myeloencephalopathy Outbreak

### **Gillian Perkins** ^1^; Bettina Wagner^2^, DVM, PhD; Alicia Rollins^1^; Hanna Sfraga^1^; Erin Pearson^1^; Marta Cercone^1^

#### ^1^Cornell University, Ithaca, NY, USA; ^2^Professor, Population Medicine and Diagnostic Sciences, Cornell University, Ithaca, NY, USA

**BACKGROUND:** Two experimental EHV‐1 challenge studies showed moderate serum antibody concentrations paired with a rapid increase in nasal mucosal anti‐EHV antibodies (mucAbs) prevent infection and viral shedding. Measuring mucAbs in an EHV‐1 outbreak may allow for reduced quarantine time for in‐contact animals that are protected.

**HYPOTHESIS/OBJECTIVES:** Our objective was to apply this test to a naturally occurring EHV‐1 incident. We hypothesized that combined PCR and EHV‐1 antibody testing could identify exposure in non‐clinical horses during an outbreak.

**ANIMALS:** Two horses with neurological signs from one farm admitted to an equine hospital. EHV‐1 was confirmed by PCR on a nasal swab in one horse. Five concurrently hospitalized horses that were previously vaccinated against EHV‐1/4.

**METHODS:** Descriptive longitudinal study. In‐contact horses were monitored for clinical signs. Serum and nasal swab samples were taken for EHV‐1 PCR and antibody quantification (EHV‐1 Risk Evaluation Assay, Cornell University) between days 6 to 21 of potential EHV‐1 exposure.

**RESULTS:** None of the in‐contact horses developed fever or clinical signs. All PCR results were negative. Only mild seroconversion was observed. MucAbs were initially low and increased rapidly in four in‐contact horses who were considered exposed to EHV‐1 yet neither infected nor infectious. One horse without an increase in mucAbs was not exposed.

**CONCLUSIONS/CLINICAL IMPORTANCE:** MucAb testing and PCR should be performed in potentially exposed horses as soon as the EHM index case is confirmed. MucAbs provide information on EHV‐1 exposure and, together with clinical monitoring and PCR, enable improved management of EHM outbreaks.

## Comparison of Duodenal Biopsies and Organoids from Healthy Horses with and Without Insulin Resistance

### Breanna Sheahan^1^; Jillian Petersen^2^; Lara Madding^3^, PhD

#### ^1^College of Veterinary Medicine, North Carolina State University, Raleigh, NC, USA; ^2^Veterinary Student, North Carolina State University, Raleigh, NC, USA; ^3^Research Coordinator, North Carolina State University, Raleigh, NC, USA

**BACKGROUND:** Equine metabolic syndrome (EMS), characterized by insulin resistance (IR), may be associated with altered enterocyte glucose absorption or incretin expression (GI hormones). Intestinal organoids are utilized for understanding epithelial biology and disease in human medicine. Organoids may be used to investigate intestine‐specific contributions to EMS. However, the alteration of relevant cellular markers in equine organoid culture has not been explored.

**OBJECTIVE:** To compare duodenal organoids and biopsies from healthy horses with and without IR.

**ANIMALS:** 12 healthy adult NC State‐ or client‐owned horses (age: 3‐22 years, 6 mares/6 geldings). All horses were maintained on pasture and biopsied during April‐July.

**METHODS:** After fasting, an Oral Sugar Test (OST; 0.45 ml/kg Karo syrup) was performed. Horses were categorized as IR based on [insulin] at 60 or 90 minutes post‐OST. Gastroscopy was performed to obtain duodenal biopsies, which were used for qRT‐PCR, immunofluorescence, or duodenal organoid culture. After 5‐7 days growth, organoids were used for qRT‐PCR or immunofluorescence. Gene expression for representative enterocyte genes was compared between tissue and organoids.

**RESULTS:** 5/12 (41%) horses were characterized as IR. IR horses trended towards increased SGLT1 expression in biopsies (Figure 1). Organoid culture caused downregulation of several genes (GIP, GCG, SGLT1, MUC2, LGR5) and upregulation of OLFM4 and LYZ (Figure 1, 2). There was no change in CHGA expression.

**CONCLUSIONS AND CLINICAL IMPORTANCE:** Duodenal organoids have altered gene expression compared to biopsies and this should be considered when studying intestinal disease in vitro. Increased duodenal SGLT1 expression may be associated with EMS independent of diet.

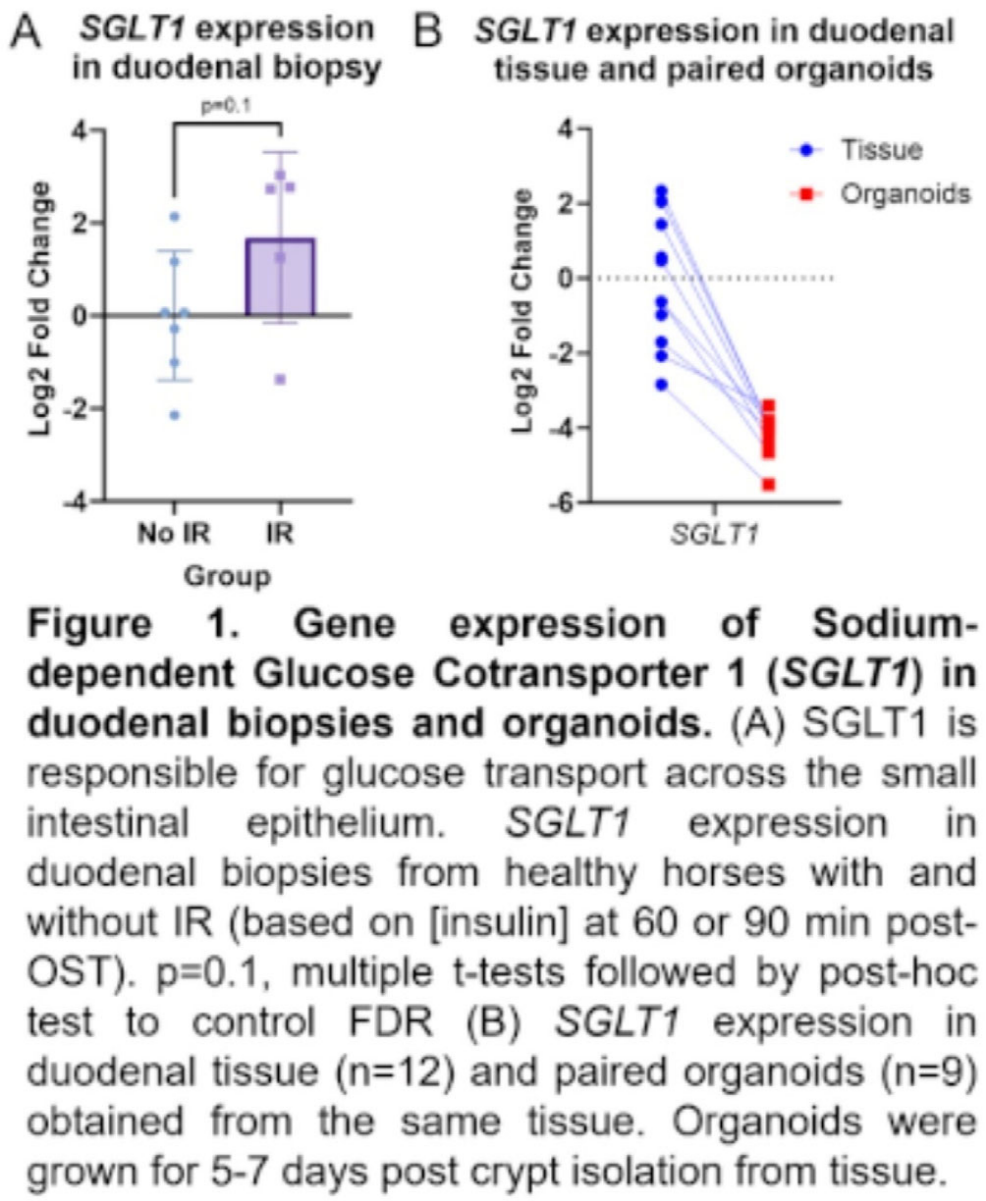

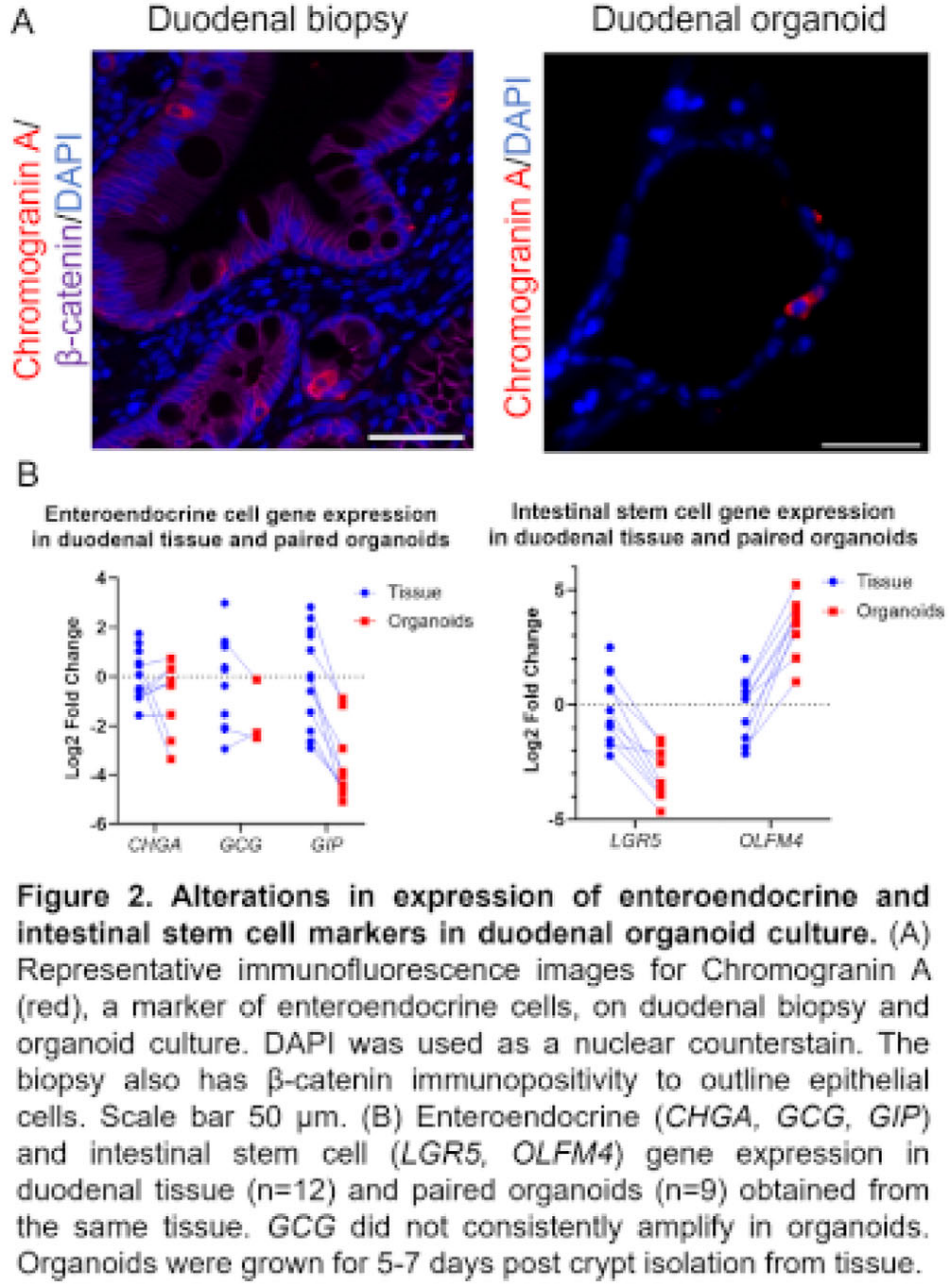

## Distribution of Alprazolam into the Milk of Lactating Mares and Subsequent Absorption by Nursing Foals

### **Camilla Quattrini** ^1^; Heather Knych^2^, DVM, PhD, DACVCP; Gary Magdesian^2^, DVM, DACVIM (LAIM), DACVECC, DACVCP

#### ^1^University of Georgia, Athens, GA, USA; ^2^University of California‐Davis, Davis, CA, USA

**BACKGROUND:** Alprazolam is a benzodiazepine used to facilitate mare‐foal bonding in aggressive or anxious postpartum mares. In humans alprazolam crosses the blood‐milk barrier, but the amount transferred into milk is minor and compatible with breastfeeding; the relative infant dose is <10%. Similar data are not available for horses.

**HYPOTHESIS/OBJECTIVES:** To measure alprazolam in serum and milk of mares (milk: serum ratio) administered alprazolam, and to determine serum concentrations of alprazolam in nursing foals to estimate extent of absorption.

**ANIMALS:** 7 healthy post‐partum mares and foals.

**METHODS:** Prospective observational study. Mares received alprazolam (0.04 mg/kg PO, q12h) for 6 days. Venous blood and milk samples were collected on days 3, 4, 5, and 6, just before the next dose, and were used to calculate milk: serum ratios and estimate the extent of absorption of alprazolam by foals. A validated liquid chromatography‐tandem mass spectrometry assay was used to measure alprazolam and its metabolite, α‐hydroxyalprazolam.

**RESULTS:** There were no significant differences in concentrations of alprazolam or α‐hydroxyalprazolam in mare serum, milk, or foal serum over time. Milk:serum ratios were similar to or higher than those reported in humans (median:0.64; range:0.42‐3.0). Relative foal dose (RFD) based on 12 h concentrations was <10% in all foals and in 96% of total samples. Foal serum concentrations of alprazolam were 6.6 ± 4.1% of those in mare serum at the same time points.

**CONCLUSIONS AND CLINICAL IMPORTANCE:** Milk:serum ratios of alprazolam in mares are variable. Foal serum concentrations and RFD suggest that alprazolam is safe for use in mares with nursing foals.

## Heart Rate Variability Associated with Endotoxin Administration in 13 Adult Horses

### **Elizabeth Williams Louie**; Marta Cercone; Barbara Delvescovo; Katharyn Mitchell

#### Cornell University, Ithaca, NY, USA

**BACKGROUND:** Horses with endotoxemia often have persistent tachycardia +/− arrhythmias. Heart rate variability (HRV) decreases in other species following endotoxin administration but has not been documented in horses.

**OBJECTIVES:** Quantify the changes in HRV following low‐dose endotoxin administration.

**ANIMALS:** Thirteen adult, systemically healthy horses were enrolled with normal physical examination, hematology, biochemistry, cardiac troponin, echocardiography, and ECG.

**METHODS:** Horses were administered *E. coli* LPS 30 ng/kg IV. Echocardiography and 24‐hour Holter ECG were performed. Treatment with intravenous fluids, polymyxin B, and flunixin meglumine was administered at 5 hours. Frequency and type of arrhythmias were documented. Time, frequency, and nonlinear domain HRV analysis were performed before, during, and after endotoxin administration.

**RESULTS:** Before treatment (time 0‐5 hours) 11/13 horses had atrial premature complexes, 6/13 had ventricular premature complexes and 7/13 had sinus block or sinus arrest. Compared to the entire Holter, during the first hour following endotoxin administration the Parasympathetic Nervous System Index decreased and the Sympathetic Nervous System (SNS) Index increased (mean difference: 1.44 and − 1.22 respectively, *P* < 0.0001 and *P* < 0.0001) indicating SNS stimulation. Indices of overall HRV showed significant differences, the Standard Deviation of Normal‐Normal intervals was decreased at 3‐5 hour (Mean difference: 65, *P* = 0.0239) and the Triangular index of NN intervals was significantly decreased at 0‐1, 1‐3 and 3‐5 hour (Mean hdifference: 1201, 1019, 1284, *P* = 0.0032, 0.0145, and 0.0017 respectively). Indices of short‐term variability were not different.

**CONCLUSIONS AND CLINICAL IMPORTANCE:** Low‐dose endotoxin administration causes arrhythmias and SNS stimulation. Analysis of HRV could be considered in monitoring cases of clinical endotoxemia.

## Omega‐3 Polyunsaturated Fatty Acids Supplementation Modulates Airway Response to Dust Exposure in Thoroughbred Racehorses

### **Laurent Couetil** ^1^; Kathleen Ivester^2^, DVM, PhD, DACVS; Laura Murray^3^, RVT; Ryan Carpenter^4^, DVM, MS, DACVS(LA); Scott Hay^5^, DVM; Clayton McCook^6^ , DVM, MS; John Burgess^7^, PhD; Thomas Peters^8^, PhD; Jae Hong Park^9^, PhD

#### ^1^Purdue University, West Lafayette, IN, USA; ^2^Research Scientist, Veterinary Clinical Sciences, Purdue University, West Lafayette, IN, USA; ^3^Laboratory Manager, Veterinary Clinical Sciences, Purdue University, West Lafayette, IN, USA; ^4^Surgeon, Private Practice, Equine Medical Center; ^5^President, Managing Shareholder, Private Practice, TFB Equine, Fort Lauderdale, FL, USA; ^6^Veterinarian, Private Practice, Equine Sports Medicine & Surgery; ^7^Associate Professor, Nutrition Science, Purdue University, West Lafayette, IN, USA; ^8^Occupational and Environmental Health, University of Iowa, Iowa City, IA, USA; ^9^Associate Professor, Occupational Health and Environmental Health Sciences, Purdue University, West Lafayette, IN, USA

**BACKGROUND:** When combined with low dust diets, omega‐3 polyunsaturated fatty acid (Omega‐3) supplementation can reduce neutrophil proportions in the bronchoalveolar lavage fluid (BAL) of horses with asthma and healthy horses. It is unknown if Omega‐3 supplementation is effective without concurrent dust mitigation.

**HYPOTHESIS/OBJECTIVES:** Dietary supplementation with fish oil rich in Omega‐3 will lower the proportion of neutrophils in the BAL of horses fed a conventional diet including hay.

**ANIMALS:** Clinically healthy racing Thoroughbreds (Nf83) housed at 4 USA racetracks.

**METHODS:** In this double‐masked controlled trial, horses were randomly assigned to receive daily fish oil (50 ml) or placebo (corn oil; 50 ml) for 4 weeks. Exposures of particulate matter smaller than 10 μm (PM10) were measured, plasma Omega‐3 concentrations were assayed and BAL was collected at baseline and after 4 weeks of supplementation. The IACUC approved all procedures. The effects of supplementation and PM10 exposure on BAL neutrophil proportions were evaluated using a generalized linear models. Significance was set at *P* < 0.05.

**RESULTS:** BAL neutrophils decreased from 8.9+/−1.2% to 3.9+/−0.9% (mean+/−s.e.m.) in horses given fish oil compared to placebo (5.9+/−1.1% to 6.1+/−1.1%; *P* = 0.014). After controlling for state and PM10 exposure, BAL neutrophils decreased as plasma Omega‐3 docosahexaenoic acid increased (*P* = 0.0005). Conversely, BAL neutrophils increased as plasma arachidonic acid increased (*P* < 0.0001).

**CONCLUSIONS AND CLINICAL IMPORTANCE:** Supplementation with fish oil rich in Omega‐3 reduces BAL neutrophil proportions in clinically healthy racehorses independent of dust exposure. Increasing Omega‐3 intake might help mitigate neutrophilic asthma in horses fed hay.

## NEUROLOGY

## Alterations in Sensory and Motor Nerve Conduction Associated with Age and Osteoarthritis in Cats

### **Aude Castel** ^1^; Aliénor Delsart^2^; Colombe Otis^3^, PhD; Manuella Lefort‐Holguin^4^; Karol‐Ann Henry^5^; Mathieu Boutin^5^; Maxim Moreau^6^; Johanne Martel‐Pelletier^7^, BSc, MS, PhD; Jean‐Pierre Pelletier^7^, MD; Eric Troncy^8^

#### ^1^Faculty of Veterinary Medicine, University of Montreal, Montreal, QC, Canada; ^2^PhD Student, Biomedecine, Research Group in Animal Pharmacology of Quebec, University of Montreal, Montreal, QC, Canada; ^3^Post‐Doc, Biomedecine, Research Group in Animal Pharmacology of Quebec, University of Montreal, Montreal, QC, Canada; ^4^Master Student, Biomedecine, Research Group in Animal Pharmacology of Quebec, University of Montreal, Montreal, QC, Canada; ^5^Neurology Resident, Clinical Sciences, Faculty of Veterinary Medicine, University of Montreal, Montreal, QC, Canada; ^6^Research Group in Animal Pharmacology of Quebec, University of Montreal, Montreal, QC, Canada; ^7^Professor, Research Group in Animal Pharmacology of Quebec, University of Montreal, Montreal, QC, Canada; ^8^Professor, Biomedecine, Research Group in Animal Pharmacology of Quebec, University of Montreal, Montreal, QC, Canada

**BACKGROUND:** Osteoarthritis (OA) leads to alteration in mobility and sensory function in elderly cats, which can be quantified through quantitative sensory testing (QST).

**HYPOTHESIS/OBJECTIVES:** Prospective, randomized comparison of nerve conduction (NC) in healthy and OA cats, to determine NC changes with health condition and its correlation with age and QST assessment.

**ANIMALS:** Research colony cats, 2 groups: 6 healthy control (CTRL) or 12 natural OA confirmed by radiographs.

**METHODS:** Under standardized general anesthesia and controlled temperature, amplitude, and conduction velocity (CV) of motor and sensory tibial and ulnar nerves were measured. Between‐group single comparisons were conducted using tests adapted to data distribution. Correlations between NC alterations, age, and QST were tested using Spearman's rank correlation.

**RESULTS:** Distal CV for both tibial (median CTRL: 97.2 vs. OA: 81.35 m.s‐1; *P* = 0.032) and ulnar motor nerves (mean CTRL: 98.6 vs. OA: 80.3 m.s‐1; *P* = 0.030), as well as amplitude for the sensory portion of both tibial (mean CTLR: 20.6 vs. OA: 12.4 μV; *P* = 0.025) and ulnar (mean CTRL: 21.6 vs. OA: 10.7 μV; *P* = 0.025) nerves, were decreased in the OA group. Negative correlation was found between age and distal CV (tibial: rho = −0.64, *P* = 0.0036; ulnar: rho = −0.48, *P* = 0.049) and sensory ulnar amplitude (rho = −0.52, *P* = 0.037). Interestingly, QST threshold was correlated with distal CV (tibial: rho = 0.56, *P* = 0.015; ulnar: rho = 0.68, *P* = 0.003), where increased nociceptive sensitization meant reduced CV.

**CONCLUSIONS AND CLINICAL IMPORTANCE:** Elderly OA cats exhibited alteration in motor and sensory NC, correlated with age and QST suggesting hypersensitization, likely contributing to impaired mobility and discomfort.

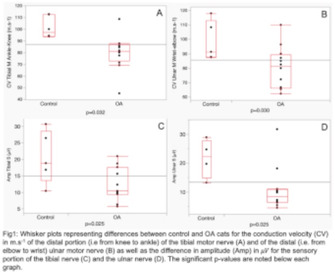

## Biomechanical Study to Assess Stabilization of Adamo Spinal Disc™ in Cervical Disc Arthroplasty Using Tetranite

### **Filippo Adamo** ^1^; Brittany McDonough^2^ ; Sourabh Boruah^2^; Molly Shutt^2^; Mike Brown^2^; Peter Wronsky^3^; Brian Hess^2^

#### ^1^East Bay Veterinary Specialists, Walnut Creek, CA, USA; ^2^RevBio, Inc., Lowell, MA, USA; ^3^RIH Orthopedic Foundation, Inc., Providence, RI, USA

**BACKGROUND:** Cervical Disc Arthroplasty (CDA) using Adamo Spinal Disc has been used as a surgical treatment in dogs affected by Disc Associated Wobbler Syndrome. Implant subsidence and loss of mobility overtime were the most frequent issues reported. The most likely cause is lack of osseointegration due to excessive micromotion at the implant‐bone interface. Tetranite is a novel synthetic‐mineral‐organic adhesive biomaterial designed specifically to adhere to metallic implants and cortical or cancellous bone and shows promise in augmenting implant stabilization during healing.

**HYPOTHESIS/OBJECTIVES:** Evaluate the immediate postoperative adhesive properties of Tetranite and its implication on CDA using the Adamo Spinal Disc.

**METHODS:** The C6‐C7 vertebral unit, from six canine cadaver cervical spines, were biomechanically tested in sub‐threshold axial compression, torsion, flexion‐extension, and lateral bending, which was then followed by axial pull‐out of the implant. Testing was sequentially performed in untreated spine (native group), treated spines with CDA without Tetranite (control group), and then treated spines with CDA with Tetranite (treatment group).

**RESULTS:** ANOVA tests indicated that the treatment group was significantly less compliant (more stable) in comparison to the native group under flexion‐extension and lateral bending, whereas the control group was not so.

**CONCLUSIONS:** Tetranite significantly improved immediate postoperative stability between the Adamo Spinal Disc and the vertebral endplate. The elimination of micro‐motion at the implant‐bone interface may facilitate osseointegration, which in turn may prevent subsidence and loss of mobility over time.

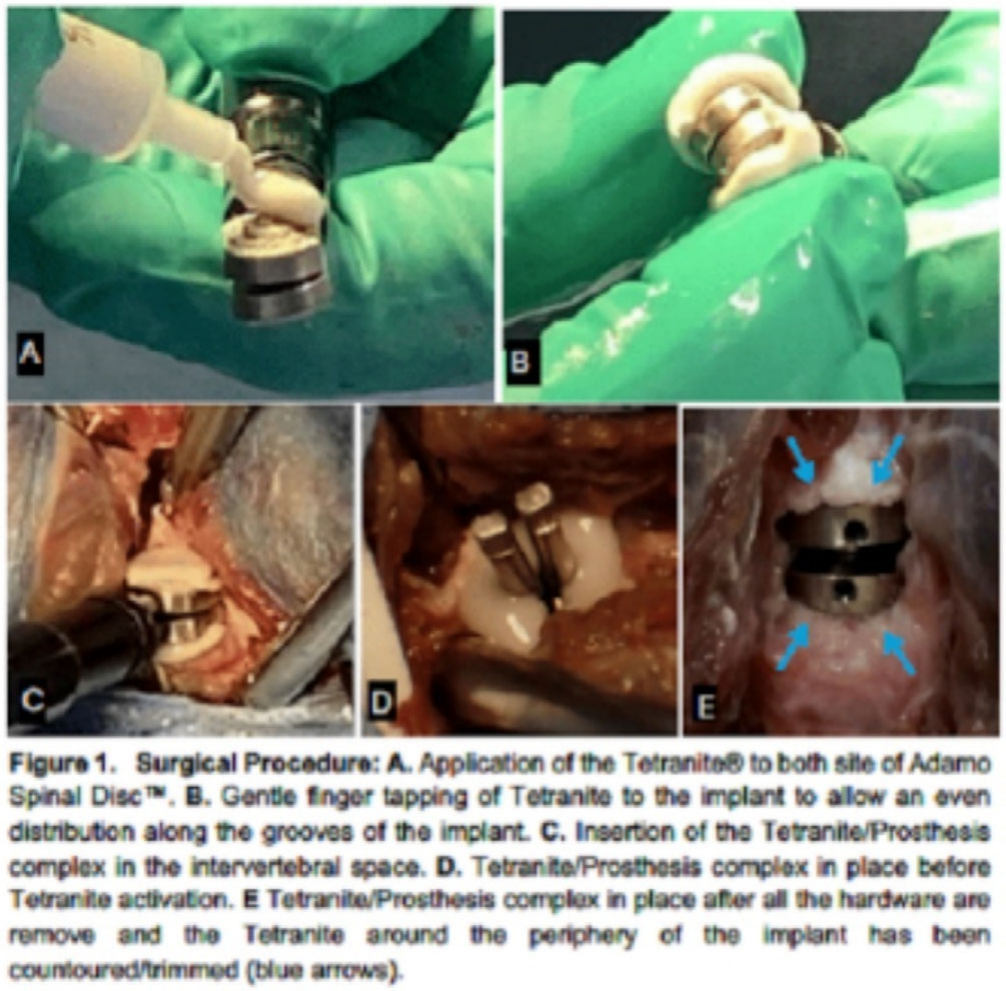

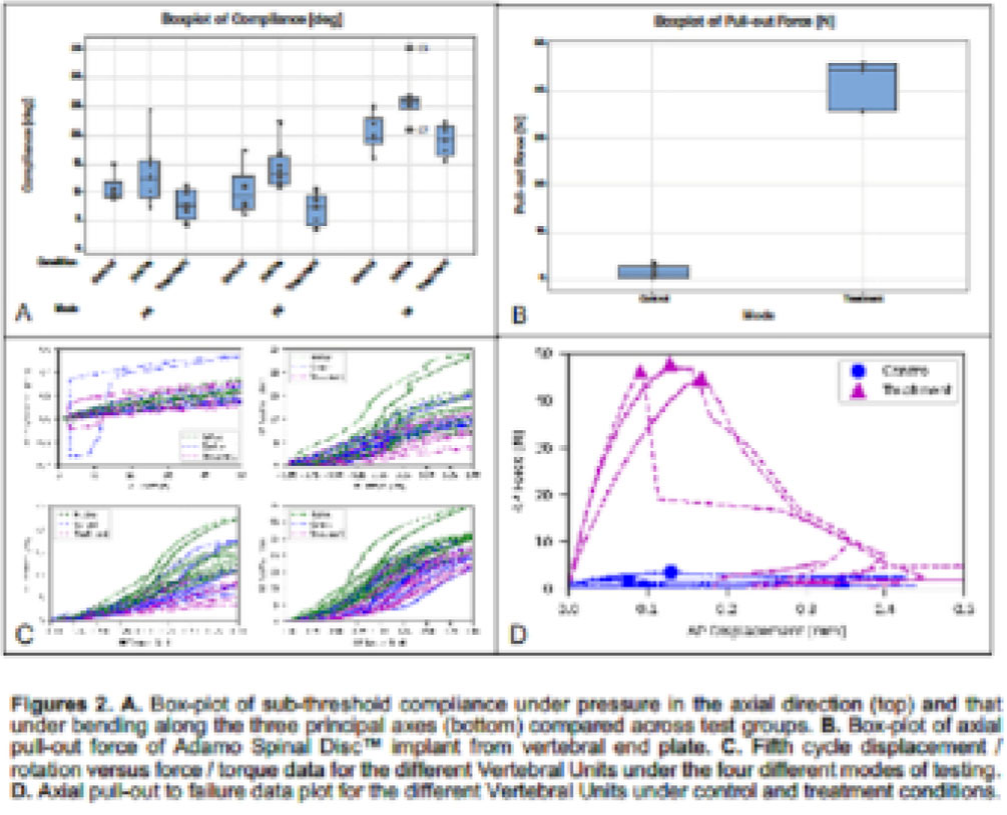

## Comparison of Stereotactic Brain Biopsy Techniques: Neuronavigation, 3D‐Printed Guides, and Neuronavigation with 3D‐Printed Guides

### **Richard Levon Shinn** ^1^; Christopher Hollingsworth^2^; Rell Parker^3^; John Rossmeisl^3^

#### ^1^Virginia Maryland College of Veterinary Medicine, Blacksburg, VA, USA; ^2^Veterinary Referral Associates; ^3^Virginia Tech, Blacksburg, VA, USA

**BACKGROUND:** To compare two previously described stereotactic brain biopsy (SBB) techniques, three‐dimensional skull contoured guides (3D‐SCGs) and neuronavigation using Brainsight, to a novel SBB technique using Brainsight combined with a 3D‐printed headframe (BS3D‐HF).

**HYPOTHESIS:** We hypothesized that the BS3D‐HF would be feasible, and more accurate than 3D‐SCGs or neuronavigation.

**STUDY DESIGN:** Prospective methods comparison.

**SAMPLE POPULATIONS:** Five canine cadavers.

**METHODS:** Initial CT was performed on cadavers with fiducial markers in place. Ten different target points were randomly selected for each method. The headframe for the BS3D‐HF was designed and printed. Trajectories were planned for each method. Steinmann pins (SPs) were placed into the target points using the planned trajectories for each method, and CT was repeated. Accuracy was assessed by overlaying the initial CT onto the post CT, and measuring the difference of the planned target point to the SP placement.

**RESULTS:** For 3D‐SCG, the median deviation was 1.32 mm (0.37‐2.22). With neuronavigation, the median deviation was 1.64 mm (0.49‐2.56). For BS3D‐HF, the median deviation was 7.13 mm (4.8‐11.1). There was no significant difference between 3D‐SCG and neuronavigation for the median deviation (*P* = 0.13). When comparing BS3D‐HF to 3D‐SCG and BS3D‐HF, there was a significant difference in the median deviation (*P* = 0.0002).

**CONCLUSION:** Both 3D‐SCGs and neuronavigation were found to have acceptable accuracies for SBB; however, the accuracy of the BS3D‐HF technique was unacceptable for clinical applications.

**CLINICAL SIGNIFICANCE:** Although feasible, the current BS3D‐HF technique requires further refinement before it can be recommended for use for SBB in dogs.

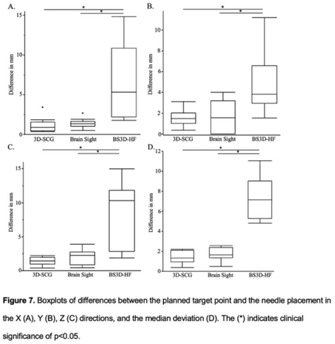

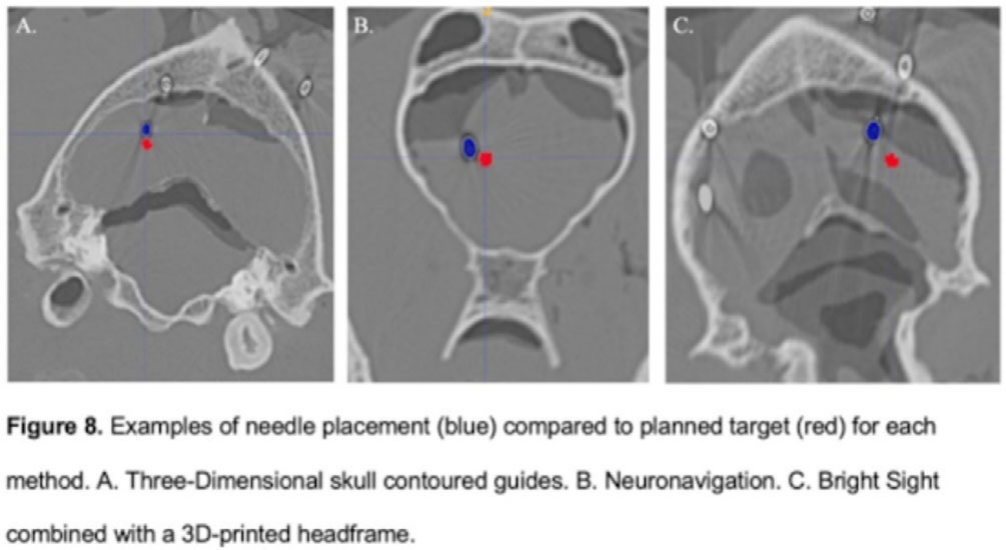

## Disposition of a Single Oral Dose of the Novel Antiseizure Drug Perampanel in Cats

### **Fiona M. Inglis** ^1^; Dawn Boothe^2^, DVM, PhD, DACVIM (Internal Medicine), DACVCP; Tom Jukier^3^, DVM, MS, DACVIM (Neurology)

#### ^1^Blue Pearl Veterinary Hospital, Englewood, CO, USA; ^2^Alumni Professor, Anatomy, Physiology, Pharmacology, College of Veterinary Medicine, Auburn University, Auburn, AL, USA; ^3^Assistant Professor, College of Veterinary Medicine, Auburn University, Auburn, AL, USA

**BACKGROUND:** Seizures represent a common neurological cause for hospitalization in cats. To date, however, choices of anti‐seizure drugs for cats are limited due to adverse reactions or to inconvenient dosing schedules. Perampanel, a highly selective, non‐competitive antagonist of the AMPA subtype of glutamate receptor, is a recently approved anti‐seizure medication in humans with a novel mechanism of action.

**OBJECTIVE:** Determine the disposition of a single dose of oral perampanel in healthy cats.

**ANIMALS:** Six healthy cats drawn from a research colony.

**METHODS:** A 0.3 mg/kg dose of perampanel was administered orally. Blood samples were collected over 72 hours, and plasma perampanel concentration was quantified using high‐pressure liquid chromatography. Data were subjected to non‐compartmental analysis.

**RESULTS:** Median (min‐max) of maximal concentration, last concentration, time to maximal concentration, last time point, disappearance half‐life, and area under the time concentration curve were 52 (38‐106) ng/mL, 20 (18‐25) ng/mL 1.5 (1‐10) hours, 36 (12‐48) hours, 22 (13‐268) hours, and 2117 (793‐8648) ng*h/mL. All but one cat failed to reach concentrations considered to be therapeutic in humans (100‐1000 ng/mL). All cats appeared to have tolerated a single dose of perampanel.

**CONCLUSIONS AND CLINICAL IMPORTANCE:** A single 0.3 mg/kg dose of perampanel appears to be safe in cats. The half‐life may be sufficiently long to allow a q12h dosing interval. However, a dose adjustment may be necessary to achieve concentrations within the human reference interval.

## Teleneurology in Veterinary Medicine for Neurolocalization

### **Richard Levon Shinn** ^1^; Brett Connolly^2^

#### ^1^Virginia Maryland College of Veterinary Medicine, Blacksburg, VA, USA; ^2^Virginia Tech, Blacksburg, VA, USA

**BACKGROUND:** Telemedicine is becoming increasingly utilized within veterinary medicine since the COVID‐19 pandemic's social distancing mandates. The validity and accuracy of telemedicine specifically for neurolocalization has yet to be investigated in the veterinary medicine.

**HYPOTHESIS:** Neuroanatomic lesion localization derived by teleneurology would have strong agreement with the in‐person neurolocalization. Our secondary hypothesis was that review of video recorded neurological examinations would have less agreement compared to live‐streamed neurological examinations.

**ANIMALS:** 55 client‐owned animals presenting to the neurology service.

**METHODS:** Prospective cross‐sectional study. Veterinary students on the neurology service performed a neurological examination. The examination was either recorded and viewed later by a neurologist, or performed and live streamed while viewed by a neurologist who was able to communicate with the student. All animals were then examined by either a neurologist or third year neurology resident blinded to the teleneurology results.

**RESULTS:** A total of 30 dogs had recorded and 25 had live streamed neurological examinations. Both recorded (k = 0.85, p [sic]).

**CONCLUSIONS AND CLINICAL RELEVANCE:** The strong correlation for both recorded and live streamed neurological examinations show promise in neurolocalization with teleneurology. Further evaluation of accuracy with pet owners and primary care veterinarians is warranted.

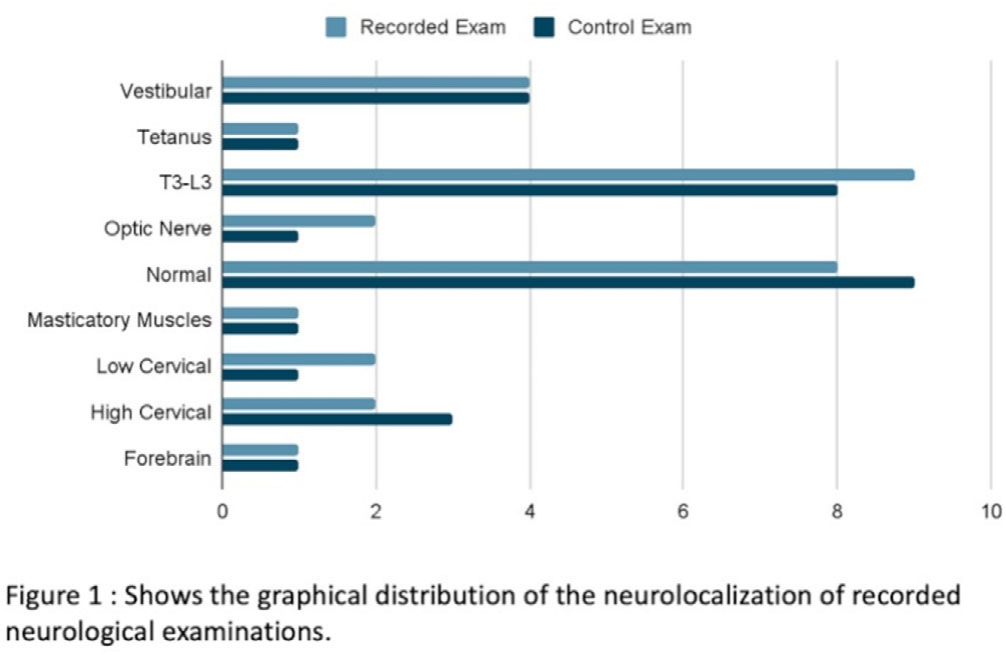

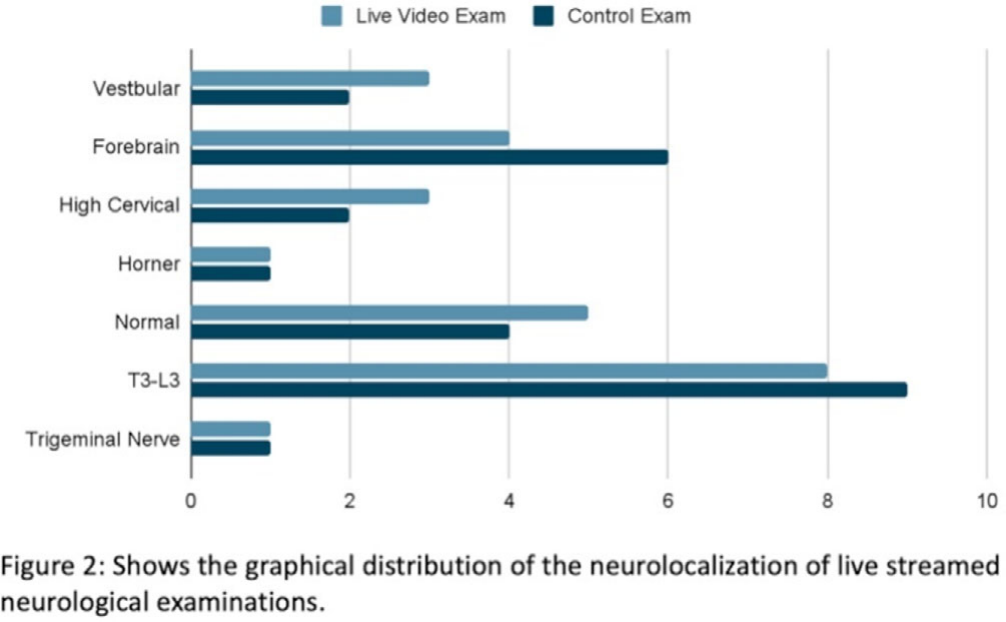

## NUTRITION

## Correlation Between Taurine Concentrations in Skeletal and Cardiac Muscle in Cadavers of Large Dogs

### Robert C. Backus

#### University of Missouri

**ABSTRACT**

**BACKGROUND:** Dilated cardiomyopathy is attributed to taurine deficiency when low blood taurine concentration is found. Nevertheless, skeletal muscle depletes of taurine before blood taurine when diets low in bioavailable sulfur‐containing amino acids are given.

**HYPOTHESIS/OBJECTIVES:** To assess whether skeletal and cardiac muscle taurine concentrations are correlated, taurine concentrations in semimembranosus muscle and left ventricular free‐wall of dog cadavers were determined.

**ANIMALS:** Cadavers of 27 males (21‐38 kg) used in student surgery laboratories were studied. They were obtained from animal shelters, preserved by freezing immediately after euthanasia, and dissected after thawing 3 days at 4°C, then 10 hours at 21‐23°C.

**METHODS:** As taurine is not readily degraded in tissues, portions of thawed muscle (0.5 g) were diluted and homogenized in water spiked with internal standard and assayed for taurine content with a validated HPLC‐UV phenyl isothiocyanate method.

**RESULTS:** Taurine concentrations (median [range]) in skeletal muscle were greater (*P* < 0.01) than those in cardiac muscle (23 [14‐34] vs. 17 [11‐34] μmol/g). The concentrations were not significantly correlated between the tissues (ρ = −0.22, *P* = 0.26). Cardiac but not skeletal muscle in dogs of the lowest body weight quartile (n = 6, <25 kg) had lower taurine concentrations relative to the other dogs (*P* < 0.01).

**CONCLUSIONS AND CLINICAL IMPORTANCE:** The findings add to the little available data on canine cardiac taurine concentrations and indicate taurine depletion in cardiac muscle of dogs may not be well reflected in taurine concentrations in skeletal muscle, which is more accessible for biopsy than myocardium.

## Dietary Response of Dogs with Protein Losing Enteropathy: A Prospective, Randomized, Blinded Multicenter Clinical Study

### **Sally C. Perea** ^1^; Isabelle Ruhnke^2^, MedVet, DrMedVet, MSc, PhD, DECVCN (Nutrition); Valérie Freiche^3^, DMV, PhD, Spécialiste en Médecine Interne (DESV‐MI); Paul Remmel^3^, DVM; Juan Hernandez^4^, DVM, DACVIM (SAIM), PhD; Amandine Drut^5^; Jeremy Laxalde^6^; Yann Queau^6^, DVM, DACVIM (Nutrition); Vincent Biourge^6^, DVM, PhD, DACVIM (Nutrition)

#### ^1^Royal Canin; ^2^Tierklinikum Freie Universität Berlin; ^3^Internal Medicine, Ecole Nationale Vétérinaire d'Alfort; ^4^PR, Clinical Sciences, Oniris VetAgroBio Nantes; ^5^Oniris VetAgroBio Nantes; ^6^Royal Canin SAS

**ABSTRACT**

**BACKGROUND:** Nutritional support of animals with Protein‐losing enteropathy (PLE) is essential to compensate energy and protein losses. However, clinical evidence regarding precise type of diet is limited.

**OBJECTIVE:** To compare the clinical response of PLE dogs fed a low‐fat diet or a combined hydrolysed, low‐fat diet while receiving medical therapy.

**ANIMALS:** Twenty‐six, client‐owned dogs diagnosed with PLE were included. Exclusion criteria covered comorbidities/metabolic diseases.

**METHODS:** Dogs were randomly assigned to a control (n = 14, commercial 22% protein, 7% fat, as fed, 3450 kcal/kg) or test diet (n = 12, 25% hydrolyzed‐protein, 7% fat, as fed, 3518 kcal/kg) and clinically evaluated at 0, 1, 3, 5, 10, 14, and 24 weeks. All dogs received veterinary care which included symptomatic treatment, immunosuppressives, antibiotics, and/or probiotics as determined by the veterinarian specialist. A Linear Mixed Model with diet and time and their respective interactions as fixed effects and dog as random term was used, significance set at *P* < 0.05. Drop‐out rates were analyzed using Fisher's Exact Test.

**RESULTS:** Ten dogs completed the study. No statistical difference was seen between the diets for drop‐out probability, body weight, Canine Inflammatory Bowel Activity Index (CIBDAI), Canine Chronic Enteropathy Clinical Activity Index (CCECAI), or albuminemia. However, time had a significant impact on CIBDAI & CCECAI scores (*P* < 0.05).

**CONCLUSION AND CLINICAL IMPORTANCE:** Dogs that completed the study improved their Clinical Activity indices under both dietary regimens. This can be associated with a better clinical outcome.

## SMALL ANIMAL INTERNAL MEDICINE

## Biological Variation of Serum Phosphorus and Fibroblast Growth Factor‐23 in Cats with Chronic Kidney Disease

### **Stacie C. Summers** ^1^; Donald Szlosek^2^; Helen Michael^2^; Rebekah Mack^2^

#### ^1^Oregon State University; ^2^IDEXX Laboratories, Inc.

**ABSTRACT**

**BACKGROUND:** Biological variation parameters are useful for choosing a subject‐ or population‐based reference interval and to assess the clinical relevance of changes in serial analytical measurements. Phosphorus and fibroblast growth factor‐23 (FGF‐23) are used in the diagnosis and management of phosphorus overload in cats with chronic kidney disease (CKD). Biological variation of FGF‐23 concentrations is unknown in cats.

**OBJECTIVE:** Determine the biological variation of phosphorus and FGF‐23 in healthy cats and cats with CKD.

**ANIMALS:** 11 healthy client‐owned cats and 8 client‐owned cats with stable CKD Stage 1‐3.

**METHODS:** Serum samples were collected once weekly for 6 weeks and frozen for batch analysis in duplicate at a commercial laboratory. Restricted maximum likelihood estimations were used to determine intraindividual (CVI), between individual (CVG), and analytical coefficients of variation (CVA) and then used to calculate the inverse index of individuality (II) and bidirectional reference change value (RCV) for serum phosphorus and FGF‐23 concentrations.

**RESULTS:** CVA for serum phosphorus concentrations was 0.9% and for serum FGF‐23 concentrations was 12.4%.

**CONCLUSIONS AND CLINICAL IMPORTANCE:** Overall, biological variation estimates for each analyte were similar between healthy cats and those with CKD. Phosphorus has low to intermediate individuality, indicating that a population‐based reference interval is appropriate. FGF‐23 had high individuality, indicating that a subject‐based reference interval is preferred to determine the clinical relevance of concentrations over time in an individual cat with stable health status.

**Table:** TABLE. The CV_I_, CV_G_, II, and RCV for serum phosphorus and FGF‐23 concentrations in healthy cats and cats with CKD.

	CV_I_	CV_G_	Inverse II	RCV
**Healthy Cats**				
Phosphorus	9.2%	6.6%	0.7	25.5%
FGF‐23	27.2%	89.7%	3.0	82.9%
**Cats with CKD**				
Phosphorus	11.8%	10.9%	0.9	32.9%
FGF‐23	25.9%	192.4%	6.7	79.7%

## Carbapenem‐Resistant Infections in Canine Patients: A Case Series

### **Cooper Brookshire** ^1^; Taylor Hellmann^2^, PhD; Ashley Robinson^2^, PhD; Khadija Ferdous^3^, MS; Keun Seok Seo^3^, DVM, PhD

#### ^1^College of Veterinary Medicine, Mississippi State University; ^2^University of Mississippi Medical Center; ^3^Mississippi State University

**ABSTRACT**

**BACKGROUND:** Carbapenem‐resistant Enterobacterales (CRE) are an emerging threat in veterinary medicine, resistant to nearly all antibiotics, transmissible within veterinary hospitals, associated with high mortality rates, and likely zoonotic pathogens.

**HYPOTHESIS/OBJECTIVES:** Our primary objectives include analyzing clinical history, identifying mechanisms of resistance, and proposing hypotheses for future studies to develop tools for CRE prevention and management.

**ANIMALS:** This study includes an analysis of six CRE infections in dogs.

**METHODS:** A case series was compiled outlining clinical history, management, and outcomes. Whole genome sequencing was performed on a subset of isolates to elucidate resistance mechanisms, strain types, and strain relationships.

**RESULTS:** CRE species included *Klebsiella pneumoniae*, *Escherichia coli*, and *Enterobacter cloacae*. CRE infections were associated with a high mortality rate; 5/6 patients were euthanized due to clinical deterioration. 5/6 were prescribed enrofloxacin and a beta‐lactam antibiotic in the three months preceding the CRE diagnosis. Clinical history analysis suggests that 4/6 patients had a hospital acquired infection. The NDM‐5 carbapenemase resistance gene was detected in the three isolates submitted for sequencing and was extremely conserved between the isolates.

**CONCLUSIONS AND CLINICAL IMPORTANCE:** CRE presents a complex and urgent challenge in veterinary medicine. The findings of this case series suggest a potential link between antibiotic therapy involving fluoroquinolone plus beta‐lactam antibiotics and CRE infections, which justifies future studies to explore and validate this hypothesis. Furthermore, CRE appears to be spreading in veterinary hospitals and in the community. Comprehensive surveillance and genetic analyses are essential for understanding CRE epidemiology in veterinary species.

## Comparison of Next‐Generation DNA Sequencing and Bacterial Culture in Bile from Dogs and Cats

### **Danielle Rondeau** ^1^; Janina Krumbeck^2^, PhD

#### ^1^Maine Veterinary Internal Medicine; ^2^MiDog, LLC

**ABSTRACT**

**BACKGROUND:** Bacterial infection is a common cause of hepatobiliary disease in small animals. The utility of next‐generation sequencing (NGS) on bile hasn't yet been fully defined.

**HYPOTHESIS/OBJECTIVES:** To compare the biliary transcriptome to results of bacterial culture in dogs and cats with hepatobiliary disease. We hypothesized that NGS would have a higher sensitivity for detecting the presence of bacteria.

**ANIMALS:** Twenty‐six client‐owned animals (17 dogs and 9 cats) that had bile culture submitted where additional bile was available for NGS.

**METHODS:** Bile was collected by ultrasound‐guided cholecystocentesis or surgical laparotomy/laparoscopy. Aerobic culture was performed on all samples, and most (22/26 samples) had anaerobic culture. NGS samples were stored frozen in a DNA stabilization solution immediately after collection and were processed upon conclusion of all sample collection. Descriptive statistics were utilized.

**RESULTS:** Fifteen percent (4/26) of samples had positive cultures, and 75% (3/4) of these yielded multiple bacterial species. All but one cultured bacterial species (scant *Micrococcus*) were also detected using NGS. In one cat with positive aerobic (*E. coli*, *Streptococcus*) and anaerobic (*Bacteroides*) cultures, NGS detected these species and multiple additional species (*Fusobacterium*, *Fretibacterium*, *Desulfomicrobium*, *Schaalia*, Lactobacillales, *Tannerella*, and *Campylobacter*) in high abundance. NGS did not reveal a high bacterial abundance (>100 000 cells/sample) in any culture‐negative sample.

**CONCLUSIONS AND CLINICAL IMPORTANCE:** NGS may be a clinically useful tool in detecting bacteria present in bile samples obtained from dogs and cats, particularly in cases where multiple species of bacteria and/or anaerobic infection is present.

## Effects of Paricalcitol on Renal Secondary Hyperparathyroidism and Proteinuria in Dogs with CKD

### **Hilla Chen**; Gilad Segev, DVM, DECVIM‐CA; Michal Mazaki‐Tovi, DVM, DECVIM‐CA

#### Koret School of Veterinary Medicine, The Hebrew University of Jerusalem

**ABSTRACT**

**BACKGROUND:** Renal secondary hyperparathyroidism (RHPT) occurs in dogs with chronic kidney disease (CKD). Paricalcitol, a 2nd generation vitamin D analog may have beneficial effects including PTH reduction, proteinuria attenuation, and prolonged survival.

**HYPOTHESIS/OBJECTIVES:** Determine the effect of paricalcitol on RHPT and proteinuria in dogs with CKD.

**ANIMALS:** Thirteen dogs with naturally‐acquired CKD stage 2‐4.

**METHODS:** Double‐blinded, placebo‐controlled study. Dogs were randomly allocated to receive placebo or paricalcitol (14 ng/kg PO once daily) in a crossover design of two, twelve‐weeks arms. Dogs were evaluated every 3 weeks. Associations between treatment, visit and the outcome variables were assessed using generalized estimating equations. Separate analyses were performed for each variable level for which an interaction effect was observed.

**RESULTS:** There was no difference in base‐line creatinine of paricalcitol‐ and placebo‐treated dogs (2.87 mg/dL, 2.25‐3.48 vs. 2.81 mg/dL, 2.16‐3.45, *P* = 0.603). With each visit, PTH concentrations decreased by 22% (7‐35%, *P* = 0.006) in the paricalcitol‐treated dogs, and increased by 18% (2‐37%, *P* = 0.022) in the placebo‐treated dogs. FGF‐23 concentrations at 12 weeks increased compared to baseline in the paricalcitol‐treated dogs (6941 pg/mL, 1781‐20 057 vs. 489 pg/mL, 188‐1272, *P* < 0.001, respectively), but not in the placebo‐treated dogs (696 pg/mL, 316‐1531 vs. 955 pg/mL, 308‐2963, *P* = 0.529). Urine protein‐to‐creatinine ratio at 12 weeks increased compared to baseline in the placebo‐treated dogs (0.8, 0.3‐1.3 vs. 0.5, 0.2‐0.9, *P* = 0.040, respectively), but not in the paricalcitol‐treated dogs (0.6, 0.3‐0.9 vs. 1.0, 0.1‐1.8, *P* = 0.351). Ionized calcium did not change between visits in both groups.

**CONCLUSIONS AND CLINICAL IMPORTANCE:** Paricalcitol attenuated RHPT in dogs with CKD.

## Evaluation of IL‐2 Suppression and Cyclosporine Concentrations in Dogs with Immune‐Mediated Chronic Hepatitis

### **Tarini V. Ullal** ^1^; Sarah Shropshire^2^, DVM, PhD, DACVIM (SAIM); Andrew Mackin^3^, BVMS, MVS, DVS, DACVIM; Todd Archer^4^, DVM, MS, DACVIM; Lakshmi Narayanan^5^; Yi Chang^5^; David Twedt^6^, DVM, DACVIM (SAIM)

#### ^1^University of California, Davis; ^2^Assistant Professor, Colorado State University; ^3^Professor, Mississippi State University; ^4^Assistant Professor, Mississippi State University; ^5^Mississippi State University; ^6^Professor Emeritus, Colorado State University

**ABSTRACT**

**BACKGROUND:** Measurement of IL‐2 suppression and cyclosporine concentrations are methods to monitor efficacy of cyclosporine therapy in dogs.

**OBJECTIVES:** Determine magnitude of IL‐2 suppression and cyclosporine concentrations in dogs receiving modified cyclosporine for treatment of immune‐mediated chronic hepatitis (ICH). Determine if cyclosporine achieves target IL‐2 suppression, cyclosporine concentrations, and biochemical and histopathologic remission of ICH.

**ANIMALS:** Nineteen client‐owned dogs with ICH.

**METHODS:** Dogs were treated with cyclosporine (starting dose of 5 mg/kg PO q 12) and monitored with serial biochemistry panels, IL‐2 suppression, and cyclosporine concentrations at 1, 3, and 6 months. Activated T‐cell expression of IL‐2 was measured via quantitative reverse transcriptase PCR. Blood trough and peak cyclosporine concentrations were measured, and target concentrations were 600 and 1400 ng/mL, respectively. Liver histopathology was re‐evaluated at 6 months. Biochemical and histopathologic remission were defined by ALT Results: All dogs had moderate/high IL‐2 suppression on at least 1 timepoint. By 6 months, mean cyclosporine dose was 7.7 mg/kg/day (range, 2‐12.5). However, 12/13 dogs showed moderate/high IL‐suppression (mean 70.8%, range 28‐88) of which 3/12 and 10/12 were in biochemical and histopathologic remission, respectively. 12/15 dogs met target trough and peak concentrations and only 2/12 dogs were not in histopathologic remission. Despite suboptimal trough concentrations in 4/16 dogs, all 4 had attained histopathologic remission.

**CONCLUSIONS AND CLINICAL IMPORTANCE:** Cyclosporine resulted in moderate/high IL‐2 suppression, target peak and trough concentrations, and histopathologic remission in the majority of dogs evaluated.

## Evaluation of Rhodanine‐Stained Liver Cytology to Diagnose and Monitor Copper‐Associated Chronic Hepatitis in Dogs

### **Tarini V. Ullal** ^1^; Sarah Shropshire^2^, DVM, PhD, DACVIM (SAIM); Russell Moore^3^, DVM, MS, DACVP; David Twedt^4^, DVM, DACVIM (SAIM)

#### ^1^University of California, Davis; ^2^Assistant Professor, Clinical Sciences, Colorado State University; ^3^Associate Professor, Microbiology, Immunology, and Pathology, Colorado State University; ^4^Professor Emeritus, Clinical Sciences, Colorado State University

**BACKGROUND:** Rhodanine‐stained liver cytology can detect hepatic copper using a minimally invasive technique and could help diagnose and monitor dogs with Copper‐Associated Chronic Hepatitis (CAH).

**OBJECTIVES:** Evaluate the utility of cytologic copper to (1) differentiate CAH from Immune‐mediated Chronic Hepatitis (ICH) and (2) monitor treatment response in CAH dogs.

**ANIMALS:** Fourteen client‐owned dogs with CAH treated with D‐penicillamine and a copper‐restricted diet for 6 months and 17 dogs with ICH.

**METHODS:** Dogs with CAH and ICH were prospectively enrolled based on histologic criteria and copper quantification. Liver cytology was graded semi‐quantitatively (1‐9) via rhodanine‐staining at diagnosis for both ICH and CAH and 1, 3, and 6 months into treatment of CAH. A Spearman's correlation was evaluated between cytologic copper grade and quantitative hepatic copper. A Wilcoxon‐rank sum test compared cytologic copper between dogs with CAH and ICH at diagnosis and pre‐ and post‐treatment in dogs with CAH.

**RESULTS:** Median (Q1‐Q3) hepatic copper (μg/g dw) was higher at diagnosis in CAH [2825 (2073‐3225)] compared to ICH [295 (24‐690)] (*P* = 0.0001) and cytologic copper grade was higher in CAH [7 (4‐7.25)] compared to ICH [4 (3.5‐6)] (*P* = 0.01). Cytologic copper grade correlated with quantitative hepatic copper in all dogs (*P* < 0.0001, rho = 0.78). In CAH dogs, hepatic copper (*P* < 0.001) and cytologic copper grade (*P* < 0.001) reduced following 6 months of chelation and dietary treatment.

**CONCLUSIONS AND CLINICAL IMPORTANCE:** Cytologic copper could help assess dogs with CAH and ICH, but there was overlap between groups and correlation with quantitative copper was moderate.

## Exenatide Extended‐Release for Maintenance of Diabetic Remission in Cats

### **Chen Gilor** ^1^; Linda Fleeman^2^; Sean Hulsebosch^3^; Stijn Niessen^4^; Charlotte Bjornvad^5^; Jully Pires^3^; Katarina Hazuchovà^6^; Jocelyn Mott^1^; Allison O'Kell^1^; Ruth Gostelow^7^; Audrey Cook^8^

#### ^1^University of Florida; ^2^Animal Diabetes Australia; ^3^University of California, Davis; ^4^Veterinary Specialist Consultations and VIN Europe; ^5^University of Copenhagen; ^6^Justus‐Liebig‐University Giessen; ^7^Royal Veterinary College; ^8^Texas A&M University

**ABSTRACT**

**BACKGROUND:** Insulin‐treated diabetic cats frequently undergo remission, but this is often temporary. The glucagon‐like peptide‐1 receptor agonist, exenatide extended‐release (exenatide‐ER) preserves and improves β‐cell function in people with type‐2 diabetes mellitus (DM).

**OBJECTIVES:** To investigate the effect of exenatide‐ER on diabetic remission in cats.

**ANIMALS:** Twenty client‐owned cats with recent (4‐12 weeks) remission of diabetes (per ALIVE criteria).

**METHODS:** In this placebo‐controlled, single‐blinded study, cats were assigned randomly to receive exenatide‐ER (0.13 mg/kg) or saline injection SC, once‐monthly for 2 years or until DM relapsed. Cats were fed low‐carbohydrate diets.

**RESULTS:** Treatment groups were similar in age, sex, and body weight upon inclusion. Fifteen cats completed the 2‐year study, and 5 cats exited it prematurely (placebo: Nf4, exenatide‐ER: Nf1). The exenatide‐ER group saw a decrease in body weight (−0.36 ± 0.5 Kg, *P* = 0.04) at study exit. In three cats, DM relapsed (placebo: Nf1, day 212; exenatide‐ER: Nf2, days 553 and 558). Excluding cats removed prematurely, the median (range) of total remission time was 745 d (742‐805) in the placebo group and 710 d (595‐763) in the exenatide‐ER group (*P* = 0.08). Hemoglobin A1c did not differ between groups (exenatide‐ER 2.5% [1.9‐5.1]; placebo 3.4% [1.8‐8.1]; *P* = 0.2) at study exit.

**CONCLUSIONS AND CLINICAL IMPORTANCE:** Exenatide‐ER contributed to weight loss but not duration of remission.

## Immunophenotype and Interleukin‐17 Expression in Dogs with Immune Mediated Hematologic Disease

### Shauna Blois^1^; Dorothee Bienzle^2^, DVM, PhD, DACVP; Benoit Cuq^3^, DVM, PhD, DACVIM (SAIM)

#### ^1^University of Guelph; ^2^Professor, Pathobiology, Ontario Veterinary College, University of Guelph; ^3^Assistant Professor, University of Dublin

**ABSTRACT**

**BACKGROUND:** Pathogenesis of canine immune mediated hematologic diseases, such as immune mediated hemolytic anemia (IMHA) and thrombocytopenia (ITP) is poorly understood. Changes noted in people with immune mediated diseases include deficient T regulatory cells, dysregulation of T cells, and excessive proinflammatory responses.

**OBJECTIVES:** The objective of this study was to describe the prevalence of selected T cell populations and interleukin (IL)‐17 producing cells in dogs with IMHA and ITP.

**ANIMALS:** Blood samples were collected prior to treatment from 15 healthy control dogs and 15 dogs with newly diagnosed non‐associative IMHA or ITP.

**METHODS:** Samples were stained with antibodies against extracellular CD4, CD8, CD25 and intracellular IL‐17 and Foxp3. Flow cytometry analysis was used to characterize lymphocytes.

**RESULTS:** Compared with healthy dogs, dogs with immune mediated hematologic disease had significantly lower frequency of CD4+ (median 17%, range 3.96‐34 versus 5.87%, 1.28‐39.3, *P* = 0.0495) and CD8+ (median 9.54%, range 3.75‐31.1 versus 0.81%, 0.19‐4.26, *P* < 0.001) lymphocytes. CD4+, CD25+, Foxp3 T regulatory cells were significantly higher in dogs with immune mediated hematologic disease compared to healthy dogs (median 1.94%, range 0.23‐13.5 versus 0.56%, 0‐7.43, *P* = 0.0028). Interleukin‐17 positivity was significantly higher in lymphocytes of dogs with immune mediated hematologic diseases compared to healthy dogs (median 0.71%, range 0.09‐4.92 versus 0.20, 0.03‐0.63; *P* = 0.0024).

**CONCLUSIONS AND CLINICAL IMPORTANCE:** A deficiency in CD4+, CD25+, Foxp3 T regulatory cells was not observed in dogs with immune mediated hematologic disease. The low frequency of CD4+ and CD8+ cells suggests T cell dysregulation in dogs with immune mediated hematologic disease. Increased production of the pro‐inflammatory cytokine IL‐17 may contribute to the pathogenesis of canine immune mediated hematologic disease.

## Neoplastic Transformation of the Colon Mucosa Correlates with an Altered Expression of RAAS in Dogs

### Giacomo Rossi^1^; Lucia Biagini^1^; Alessandra Gavazza^1^; Matteo Cerquetella^1^; Franziska dengler^2^; Romy Heilmann^3^

#### ^1^University of Camerino, Italy; ^2^Department of Veterinary Physiology, Vetmeduni; ^3^Small Animal Medicine, University of Leipzig

**ABSTRACT**

**BACKGROUND:** Tissue effects of the renin‐angiotensin‐aldosterone system (RAAS) include the regulation of cell growth and proliferation, and RAAS imbalances have been linked to cancer development and progression. RAAS components have potential as diagnostic/prognostic markers and therapeutic targets. Colonic adenomatous lesions are precursors of colorectal cancer, providing a model for carcinogenesis research.

**ANIMALS:** Archived colon tissues from 26 dogs.

**METHODS:** For each polyp (based on dysplasia/necrosis assessed as grade I: n = 10, grade II: n = 9, grade III: n = 7) and adjacent untransformed mucosa, inflammation/structural changes, goblet cells (Alcian/Pas+), mitotic index (Ki67+), apoptosis rate (TUNEL assay), and the immunohistochemical expression of the main RAAS receptors (AT1R, AT2R, and MAS1) and mineralocorticoid receptor (MCR) were scored, tested for correlations, and compared among differentiation grades.

**RESULTS:** Higher‐grade polyps had more goblet cells (*P* = 0.004) and more marked inflammation/structural lesions, proliferation, and apoptosis in the adjacent mucosa (all *P* < 0.05) than lower‐grade polyps.

**CONCLUSIONS:** Local RAAS dysregulation, with overexpression of traditional RAAS and downregulation of alternative RAAS arms, is a feature of adenomatous colonic lesions and is linked to the degree of dysplasia. Its role in angiogenesis, proliferation, epithelial‐to‐mesenchymal transition, and metastasis warrants further investigation.

## Oral Remdesivir and GS‐441524 Show Similar Efficacy in Treating Noneffusive Feline Infectious Peritonitis

### **Terza Brostoff**; Brian Murphy; Luis Diego Castillo Charpentier; Jully Pires; Amy Rose; Krystle Reagan

#### UC Davis

**ABSTRACT**

**BACKGROUND:** Untreated FIP is generally considered fatal in cats. The best‐studied antiviral drug, GS‐441524, is unlikely to reach the clinical market in the USA; the related compound remdesivir has limited published data on use for treating FIP.

**HYPOTHESIS/OBJECTIVES:** This is a clinical trial comparing overall survival between oral remdesivir and GS‐441524 to treat noneffusive FIP. We hypothesize that the rate of remission and relapse will be similar between the two treatment arms.

**ANIMALS:** Twenty client‐owned cats with confirmed spontaneous noneffusive FIP with no concurrent or previous anticoronaviral administration.

**METHODS:** This is a double‐blind, prospective, randomized clinical trial with two treatment arms. 10 cats per arm received either GS‐441524 (15‐20 mg/kg) or remdesivir (30‐40 mg/kg) PO once daily for 12 weeks. Physical examination, CBC, and chemistry were performed at 0, 6, and 16 weeks.

**RESULTS:** Eight of ten cats in the GS‐441524 and nine of ten cats in the remdesivir arm survived to 16 weeks (*P* = 0.5). Two cats in the GS‐441524 group experienced relapse at 16 weeks; relapse was not observed in the remdesivir group. The most common adverse events were GI signs, which were observed in 6 cats treated with remdesivir and 3 cats treated with GS‐441524 (*P* = 0.4).

**CONCLUSIONS AND CLINICAL IMPORTANCE:** This study represents the first direct comparison of oral remdesivir and GS‐441524 to treat noneffusive FIP. No statistical difference in survival or adverse drug reactions was observed, laying a foundation to expand clinical options in the treatment of this otherwise fatal disease.

## Prednisolone Pharmacokinetics in Dogs with Protein‐Losing Enteropathy

### **Sara Jablonski** ^1^; John Buchweitz^2^; Andreas Lehner^2^; Jessica Strohmeyer^2^; Daniel Langlois^2^

#### ^1^Michigan State University; ^2^Michigan State University College of Veterinary Medicine

**BACKGROUND:** Approximately 50% of dogs with protein‐losing enteropathy (PLE) do not improve in response to orally administered steroids. It is unknown if steroid malabsorption contributes to treatment failures.

**OBJECTIVE:** To compare pharmacokinetics of orally administered prednisolone in dogs with PLE versus healthy controls (HC).

**ANIMALS:** Fourteen dogs with PLE and 7 control dogs.

**METHODS:** Prospective case‐controlled study. Dogs treated with 1 mg/kg prednisolone PO once daily for approximately 3 weeks. Blood samples collected at set timepoints before and after prednisolone administration on first (T1 = day 1) and final (T2) study days. Total and non‐protein bound prednisolone concentrations determined with LC‐MS‐MS, and data subjected to non‐compartmental analyses. Pharmacokinetics variables compared between PLE and control dogs and between PLE short‐term responders and non‐responders.

**RESULTS:** PLE dogs had a shorter elimination half‐life (mean ± SD) than control dogs (1.4 ± 0.4 hours vs. 1.8 ± 0.3; *P* = 0.05) whereas the percentage of non‐protein bound serum prednisolone (median, interquartile range) was higher in PLE dogs versus control dogs (15.7%, 7.5%‐22.1% vs. 6.7%, 5.4‐8.8%; *P* = 0.02) at T1. Total prednisolone drug exposures and maximum total serum drug concentrations did not differ between PLE and control dogs at T1 or T2, nor did they differ between short‐term responders and non‐responders within the PLE population (*P* > 0.05 for all comparisons).

**CONCLUSIONS:** Overall drug exposures are similar between PLE dogs and healthy controls. Steroid malabsorption is unlikely to be a common cause of treatment failure in dogs with PLE.

## Retrospective Investigation into Genetic Mutations Associated with Vaccine Adverse Events

### **JoAnn Morrison** ^1^; Rebecca Foran^2^; Oliver Forman^2^; Jamie Freyer^2^; Julia Labadie^2^; Nate Spofford^1^

#### ^1^Mars Veterinary Health; ^2^Wisdom Panel

**ABSTRACT**

**BACKGROUND:** Certain dog breeds appear more likely to have adverse vaccine reactions, suggesting genetics may play a role.

**HYPOTHESIS/OBJECTIVES:** To evaluate genetic factors associated with adverse events recorded within three days of vaccination.

**ANIMALS:** 798322 dogs vaccinated at Banfield Pet Hospital from January 2016‐April 2023 that had DNA samples collected via Wisdom Panel.

**METHODS:** Electronic medical records were used to identify dogs with (cases) and without (controls) possible vaccine‐related adverse events. Control dogs were randomly selected to be five times the number of cases. Additional medical record data (eg, signalment, number of vaccines given) were collected. Genetic breed was determined using the Wisdom Panel breed detection algorithm. Dogs were genotyped using a custom 100 k Illumina Infinium XT SNP microarray and standard quality control was performed. A mixed linear model was used to calculate odds ratios and P‐values for the remaining 7506 cases and 39 072 controls, adjusting for a centered relatedness matrix using GEMMA software as well as sex, weight, and number of vaccines given. Genome‐wide significance was based on P‐value.

**RESULTS:** Two variants on chromosome 11 reached genome‐wide significance for an association with vaccine‐related adverse events (*P* = 1e‐13). The associated region includes genes encoding interleukins 4 and 13. Whole genome sequencing is currently underway to identify the locations and predicted impact of variants in this region.

**CONCLUSIONS AND CLINICAL IMPORTANCE:** Pre‐identification of dogs at increased risk for an adverse vaccine reaction could allow proactive intervention to mitigate patient risk.

## Signalment Risk Factors for Coccidioidomycosis Test Positivity in 887 764 Dogs, 2012‐2023

### **Jane E. Sykes** ^1^; Simon Camponuri^2^; Judith Escalona^1^; William Mills^3^; Ellyn Mulcahy^4^; George Thompson^1^; Amanda Weaver^2^

#### ^1^University of California‐Davis; ^2^University of California‐Berkeley; ^3^352nd Civil Affairs Command; ^4^Kansas State University

**ABSTRACT**

**BACKGROUND:** Coccidioidomycosis (“Valley fever”) is an emerging infectious disease caused by *Coccidioides* spp., soil‐borne fungi endemic to the southwestern United States. Following soil disturbance, humans and animals inhale aerosolized spores and can develop pneumonia and life‐threatening disseminated disease. Previous studies suggested an increased risk of infection among certain dog breeds (e.g., vizslas, Weimaraners, Dalmatians) and among male dogs, yet it remains unclear whether this relates to digging behavior or a true increase in susceptibility (insufficient immune response).

**HYPOTHESIS:** That analysis of a large dataset of serologic test results would shed light on signalment risk factors for *Coccidioides* infection in dogs.

**ANIMALS:** 887764 dogs tested for anti‐*Coccidioides* antibodies across 7 diagnostic laboratories (2012‐2023).

**METHODS:** Chi‐square analysis was used for statistical analysis; the group with test positivity (TP) closest to that of the total population as the reference for odds ratio (OR) calculations.

**RESULTS:** Of 887 764 dogs, 37.3% were seropositive. Sex, breed, age, and size were identified for 91%, 77%, 75%, and 64% of dogs, respectively. Breeds with the highest and lowest ORs for TP were McNabs and Shetland sheepdogs, respectively (see Table). ORs for TP were highest in large and medium sized dogs and lowest in toy sized dogs. ORs for TP were highest in neutered males and lowest in intact females. Seropositive dogs were younger than seronegative dogs; TP was highest in dogs 3 to 4 years of age.

**CONCLUSIONS AND CLINICAL IMPORTANCE:** Findings suggest that both genetic and behavioral factors may explain susceptibility to coccidioidomycosis in dogs.

**Table:** TABLE. Odds ratios for test positivity (TP) to *Coccidioides* spp. in dogs tested for *Coccidioides* spp. antibodies between 2012 and 2023 (all *P* values <0.0001).

Variable		Test positivity (TP)	OR (95% CI)	Reference group (TP)
Breed (n)	McNab	29/35 (82.9%)	7.62 (3.25‐17.85)	Greyhounds (1340/3589, [37.3%])
	Spinone Italiano	71/112 (63.4%)	2.89 (1.96‐4.26)	
	Vizsla	1852/2934 (63.1%)	2.87 (2.60‐3.18)	
	Pug	1700/8958 (19.0%)	0.39 (0.36‐0.42)	
	Pomeranian	1367/7280 (18.8%)	0.39 (0.35‐0.42)	
	Shetland sheepdog	432/2431 (17.8%)	0.36 (0.32‐0.41)	
Body Size	Large	78 674/181367 (43.4%)	1.24 (1.21‐1.27)	Giant breed dogs (14 087/36832, [38.2%])
	Medium	58 284/134762 (43.2%)	1.23 (1.20‐1.26)	
	Small	38 315/125141 (30.6%)	0.71 (0.70‐0.73)	
	Toy	22 004/90895 (24.2%)	0.51 (0.50‐0.53)	
Sex	Male neutered	113 705/292033 (38.9%)	1.1 (1.03‐1.08)	Intact male dogs (16 668/27548 [37.7%])
	Female neutered	108 482/297077 (36.5%)	0.95 (0.93‐0.97)	
	Female intact	11 122/32099 (34.6%)	0.88 (0.85‐0.90)	
Age (years)	Less than 1	8836/31938 (27.7%)	0.63 (0.61‐0.65)	Dogs 8 to 10 years (47 239/125123 [37.8%])
	1 to 3	33 857/80801 (41.9%)	1.19 (1.17‐1.21)	
	3 to 6	70 356/154652 (45.5%)	1.36 (1.36‐1.40)	
	6 to 8	42 075/97680 (43.1%)	1.25 (1.23‐1.27)	
	10 to 15	47 928/166347 (28.8%)	0.67 (0.66‐0.68)	

## Survey of Antimicrobial Stewardship and Infection Prevention and Control Efforts among US/Caribbean Veterinary Schools

### **Jennifer L. Granick** ^1^; Emma Bollig^2^, MPH; Ian DeStefano^3^ , DVM, DACVECC; Dubraska Diaz‐Campos^4^, DVM, PhD; Emily Janovyak^5^, DVM; Claire Fellman^6^, DVM, PhD, DACVIM (SAIM), DACVCP

#### ^1^College of Veterinary Medicine, University of Minnesota; ^2^Epidemiologist, Veterinary Clinical Sciences, College of Veterinary Medicine, University of Minnesota; ^3^Assistant Clinical Professor, Cummings School of Veterinary Medicine, Tufts University; ^4^Assistant Professor, College of Veterinary Medicine, The Ohio State University; ^5^USDA ORISE Fellow, College of Veterinary Medicine, The Ohio State University; ^6^Associate Professor, Cummings School of Veterinary Medicine, Tufts University

**ABSTRACT**

**BACKGROUND:** Antimicrobial stewardship (AS) and infection prevention and control (IPC) efforts within veterinary schools are largely siloed and limited in scope. Increased collaboration is needed to foster context‐appropriate AS interventions and support AS and IPC committees, professional curriculum, and practitioner/client outreach.

**OBJECTIVES:** To identify current strengths and gaps in AS and IPC among U.S. and Caribbean veterinary schools.

**ANIMALS:** N/A.

**METHODS:** Veterinary school representatives recruited via the Deans' listserv, previous AS study participants, and direct contact, were invited to take an online survey about their institution's current AS and IPC committee structure and activities.

**RESULTS:** Of 24 participating schools, 12 (50.0%) have AS and 19 (79.2%) have IPC committees. Lack of staff time (21/24, 87.5%) and dedicated resources (17/24, 70.8%) were cited as major barriers for AS, and lack of staff time as a barrier for IPC (62.5%, 15/24); 62.5% (15/24) have no dedicated faculty or staff effort for AS or IPC programs. For those without AS committees, 7/12 (58.3%) expressed uncertainty about how to establish a committee. Most schools (22/24, 91.7%) include AS in their curriculum; 4/24 (16.7%) provide training for clinical faculty. Eleven (48.5%) schools reported performing at least one activity addressing all five AVMA's AS Core Principles (Table 1); one school performed no activities.

**Table:** TABLE 1. Veterinary schools' efforts to address AVMA's Five Core Principles of Antimicrobial Stewardship.

Core principle	Number (%) of schools performing ≥1 activity; n = 24	Most common core principle activity (n, %); n = 24
Commit to AS	17 (70.8%)	‐Form AS committee (12, 50.0%) ‐Identify AS champion (12, 50.0%)
Advocate for a system of care to prevent common diseases	21 (87.5%)	‐Educate clients on importance of preventative care (19, 79.2%) ‐Design, use, and monitor detailed written IPC plans (19, 79.2%)
Select and use antimicrobial drugs judiciously	21 (87.5%)	‐Provide antimicrobial use consultations with specialist (16, 66.7%)
Evaluate antimicrobial drug use practices	18 (75.0%)	‐Engage with veterinary diagnostic laboratories to provide facility or regional antibiograms (16, 66.7%)
Educate and build expertise	17 (70.8%)	‐Provide information to clients about proper medication disposal (12, 50.0%)

AS = antimicrobial stewardship, IPC = infection prevention and control.

**CONCLUSIONS AND CLINICAL IMPORTANCE:** Human and financial resources are needed to further AS and IPC in veterinary schools. Collaboration among schools, including efforts to share resources and standardize practices, with support from national and international institutions, may enhance AS and IPC initiative feasibility.

## Ultrastructural Changes in Colonic Mucosa of Dogs with Chronic Inflammatory Enteropathy Treated with Synbiotics

### **Albert E. Jergens** ^1^; Tracey Stewart^2^, BS, MS; Dipak Sahoo^3^, PhD; Emily Lindgreen^1^; Jon Mochel^1^, DVM, PhD; Karin Allenspach^1^, DVM, PhD, DECVIM‐CA; Agnes Mochel^4^, DVM; Valerie Parker^5^, DVM, DACVIM; Adam Rudinsky^5^, DVM, DACVIM; Jenessa Winston^5^, DVM, PhD, DACVIM; Romy Heilmann^6^, DVM, PhD, DACVIM

#### ^1^Iowa State University; ^2^Microscopist, Genetics, Development, and Cell Biology, Iowa State University; ^3^Research Scientist 2, VCS, Iowa State University; ^4^University of Georgia; ^5^SA, Ohio State University; ^6^SA, University of Leipzig, Germany

**ABSTRACT**

**BACKGROUND:** Synbiotics may be used to reduce intestinal inflammation and mitigate dysbiosis in dogs with chronic inflammatory enteropathy (CIE). Previous studies have not evaluated the colonic mucosal ultrastructure of dogs with active CIE treated with synbiotics.

**AIMS:** To characterize ultrastructural changes in the colonic mucosa of dogs with CIE at diagnosis and following synbiotic treatment using transmission electron microscopy (TEM).

**ANIMALS:** Twenty‐four client‐owned dogs diagnosed with CIE were randomized to receive hydrolyzed diet (placebo [PL]) or hydrolyzed diet with synbiotic‐IgY (SYN).

**METHODS:** Endoscopic biopsies of the colon were collected, fixed in 1% paraformaldehyde and 3% glutaraldehyde in 0.1 M sodium cacodylate buffer, and routinely processed. Thick (1.5 μm) and thin (50 nm) sections were obtained using a Leica UC6 ultramicrotome. TEM images were collected using a 200 kV JEOL JSM 2100 scanning transmission electron microscope. Ultrastructural changes in microvilli length (MVL), mitochondria (MITO), and endoplasmic reticulum (ER) were compared between PL and SYN treatment groups using a two‐tailed with *P* < 0.05 considered significant.

**Student's *T*‐Test With *P* Results:** The extent of mucosal ultrastructural pathology varied in individual dogs pre‐ versus post‐treatment. While morphologic changes to enterocytes, MVL, MITO, and ER were observed, significant differences in these ultrastructural parameters were not found between PL and SYN dogs pre‐treatment. In contrast, important ultrastructural changes were observed post‐treatment with SYN‐treated dogs having significant (*P* < 0.05) ultrastructural improvement to MVL, MITO, and ER injury scores as compared to values observed in PL‐treated dogs.

**CONCLUSIONS:** Dogs with CIE exhibit colonic ultrastructural pathology that significantly improves after treatment with synbiotic‐IgY.

## Urinary Cystine/Creatinine Concentrations in Dogs with Suspected Androgen‐Dependent Cystine Urolithiasis Before and After Castration

### Jodi Westropp^1^; Larsen Jennifer^2^; Eric Johnson^2^, DVM DACVR; Pires Jully^2^; Hulsebosch Sean^2^

#### ^1^University of California; ^2^School of Veterinary Medicine, University of California‐Davis

**ABSTRACT**

**BACKGROUND:** Castration without dietary interventions might be appropriate for dogs with suspected androgen‐dependent cystinuria.

**HYPOTHESIS/OBJECTIVE:** Urinary cystine/creatinine (Ucys/crt) ratio will be significantly lower in dogs with cystine urolithiasis after castration and associated with the absence of ultrasonographic urolith recurrence.

**ANIMALS:** Intact male dogs with confirmed cystine urolithiasis.

**METHODS:** Abdominal ultrasound, urinalysis, and urine amino acid concentrations were performed at baseline (T1), one month (T2) and 3 months (T3) post castration. Genetic testing is pending. Owners were instructed to continue the dog's normal diet. If recurrence was noted at T2, dogs were transitioned to a therapeutic diet marketed for cystine prevention.

**RESULTS:** Five dogs have completed the trial: one dachshund, 2 English bulldogs, 1 French bulldog (FBD), and 1 mixed breed. At T1, all dogs had multiple uroliths removed via cystotomy and were castrated. At T2, the mixed breed and FBD had several 2‐3 mm cystoliths but no clinical signs. By T3 uroliths resolved in one dog (FBD) and improved to “scant urinary debris” in the mixed breed. Overall, UCyst/crt (nMol/mg; mean ± SD) decreased significantly over time: T1: 1082.2 ± 536.9, T2: 375.9 ± 374.9, and T3: 156.8 ± 120.4 (*P* = 0.001). In two dogs with ultrasonographic recurrence, Ucyst/crt still decreased by 82% and 66%, respectively at T2.

**CLINICAL RELEVANCE:** Ucyst/crt decreased with castration as the only intervention in 3 dogs, including the dachshund, and was associated with a lack of clinical and ultrasonographic urolithiasis. No dog required urolith removal at reevaluations. Ucyst/crt might be useful for monitoring and confirming diagnosis.

